# Comparative Transcriptome Analysis Reveals Expression of Defense Pathways and Specific Protease Inhibitor Genes in *Solanum lycopersicum* in Response to Feeding by *Tuta absoluta*

**DOI:** 10.3390/insects16020166

**Published:** 2025-02-05

**Authors:** Yan Zhou, Yongyi Pan, Jia Liu, Wenjia Yang, Guangmao Shen

**Affiliations:** 1 Key Laboratory of Entomology and Pest Control Engineering, College of Plant Protection, Southwest University, Chongqing 400715, China; yiyayiyazzz@163.com (Y.Z.); pyy202302@163.com (Y.P.);; 2 Key Laboratory of Agricultural Biosafety and Green Production of Upper Yangtze River (Ministry of Education), Southwest University, Chongqing 400715, China; 3 Key Laboratory of Surveillance and Management of Invasive Alien Species in Guizhou Education Department, College of Biological and Environmental Engineering, Guiyang University, Guiyang 550005, China

**Keywords:** *Solanum lycopersicum*, *Tuta absoluta*, host interaction, endopeptidase inhibitors, RNAseq

## Abstract

Understanding plant-insect interactions can help control the harm of herbivorous pests. According to transcriptome data, transcripts of *Solanum lycopersicum* responding to feeding by *Tuta absoluta* were screened for important endopeptidase inhibitors. These genes were annotated as serine-type endopeptidase inhibitors from the potato inhibitor I family, potato type II proteinase inhibitor family, and soybean trypsin inhibitor (Kunitz) family. Based on the analysis of expression patterns, *Solyc09g084480.2*, *Solyc03g020080.2*, *Solyc03g098760.1*, and *Solyc01g009020.1* were identified as key genes in the defense system of *S. lycopersicum*. The major endopeptidase genes such as *Tabs008250*, *Tabs007396*, and *Tabs005701* in the larval stages of *T. absoluta* were also detected as potential targets of the plant endopeptidase inhibitors. The interaction mode between these endopeptidase and endopeptidase inhibitors was predicted based on the protein structure construction. This study aims to reveal the molecular response of *S. lycopersicum* to feeding by *T. absoluta* with high throughput sequencing and bioinformatics analysis.

## Simple Summary

Plants and phytophagous insects have co-evolved for millions of years. The interaction between them includes producing defense products in plants and formation of mechanisms to resist insect attack. Understanding this process can identify active compounds and potential targets to control phytophagous insects. The tomato leaf miner *Tuta absoluta* is one of the most dangerous pests to tomato. In this study, serine-type endopeptidase inhibitors were characterized as important response factors in *Solanum lycopersicum* to resist feeding by *T. absoluta.* These data are helpful to develop new pest control compounds based on the interaction between host and pest.

## 1. Introduction

The tomato (*Solanum lycopersicum*) is one of the most important fruits. It is rich in nutrition and good for human health. The tomato leaf miner, *Tuta absoluta* (Meyrick) (Lepidoptera: Gelechiidae), is an invasive pest. Its earliest appearance can be traced back to Peru in South America in 1917, and it was introduced to Europe in 2006, subsequently becoming prevalent worldwide [1,2]. *T. absoluta* has strong reproductive capacity and each female can lay 200–300 eggs [3]. After hatching, the larvae feed on the stems, leaves, and fruits of the host plants, causing serious harm to tomato, potato, pepper, and other species of the Solanaceae family [4].

Plants and insects have co-evolved for millions of years. In this process, plants have managed to develop different defense mechanisms to resist herbivorous pests by differentiating into lignified cell walls as physical barriers or producing volatile substances and secreting secondary metabolites [5]. Jasmonic acid and salicylic acid are both related to plant insect resistance and these two pathways can also adjust each other to optimize the defense response [6,7]. Tobacco can resist insect pests by secreting alkaloids, phenylpropanoids, green leaf volatiles, terpenoids, and other chemicals [8]. The glandular trichomes on the surface of tomato leaves can produce acylsugars to resist *Bemisia tabaci* [9]. The phytic acid in the fruits and seeds of plants has a negative effect on the growth and development of *Heliothis virescens*, *Trichoplusia ni*, and *Depressaria pastinacella* [10]. Tomato plants also contain zingiberene to affect the feeding and reproductive process of *Diabrotica speciose* [11]. In addition, some toxic microorganisms are involved in plant defense, improving the resistance of plants to pests [12].

*T. absoluta* can cause tomato plants to produce herbivore-induced plant volatiles after feeding. *T. absoluta* can distinguish the volatile substances produced by *S. lycopersicum* as well. Females prefer to lay eggs on healthy and intact plants rather than those on which larvae have been feeding or those with methyl jasmonate [13]. Based on the defense mechanism of host plants, many active compounds against pests have been developed based on plant defense substances. Some plant-derived compounds, such as citrus essential oil and biogenic amide, have proven to be effective in the control of *T. absoluta* [14,15]. Clarifying the interaction between *S. lycopersicum* and *T. absoluta* would be beneficial to develop new compounds for the control of this pest.

Phytophagous insects rely on proteases to digest and absorb plant nutrients. Correspondingly, there are natural protease inhibitors (PIs) in the defense system of plants, which can target the digestive proteases of insects, thus destroying insect digestion and assimilation of food and delaying insect development [16]. In this study, transcriptome sequencing and annotation were applied to clarify the interaction between *Solanum lycopersicum* and *T. absoluta*, especially the interaction between plant PI and insect proteases.

## 2. Materials and Methods

### 2.1. Solanum lycopersicum and Tuta absoluta

The tomato *S. lycopersicum* variety JinSheng03 was used in this study. The tomato leaf miner *T. absoluta* population was obtained from the College of Biological and Environmental Engineering, Guiyang University, and this population was originally collected on *S. lycopersicum* from Yuxi city, Yunnan Province, China, and reared for more than 5 years in the laboratory. The plants and insects were continuously reared in an artificial climate chamber in Southwest University. The tomato plants were planted with nutrient soil and watered every 3 days. The rearing conditions were set as 26 ± 1 °C temperature, 60 ± 5% humidity, and a 16:8 h light/dark (L:D) photoperiod.

The adults of *T. absoluta* were reared with honey water in an insect rearing cage (50 cm × 50 cm), and tomato plants were put into the cage to collect eggs twice a day; then the plant with eggs was separately placed in another cage for the development of larvae. New plants were added for feeding by the larvae. The larvae pupated in soil under the plant and once the adults emerged, they were moved to the adult cage.

### 2.2. Sample Collection for Transcriptome Analysis

#### 2.2.1. Plant Treatment with *Tuta absoluta* Feeding and Mechanical Damage

The 5-week-old tomato plants were used for experimental treatment. The treatments were divided into mechanical damage treatment and insect feeding treatment. For the insect feeding, the three newly laid eggs at the same time were picked with a brush to the center of the leaf. Continuous observation was made to confirm that the eggs hatched at a similar time and the larvae were similar in size and that the larvae fed on the same leaf. Within 48 h after hatching, when the tunnels made by *T. absoluta* clearly appeared, the leaf was collected for RNA extraction. For the mechanical damage, the leaf was slightly rubbed with coarse sand until microdamage could be directly seen on the leaves and after 48 h, the leaf was collected for RNA extraction. The untreated leaf at the same developmental stage from a separate plant was used as the control. Three replicates were conducted for each treatment and control and all treatments and repetitions were performed on separate plants.

#### 2.2.2. Collection of Different Developmental Stages of *T. absoluta* Larva

Fresh tomato plants were used to collect eggs as described above. The eggs collected on the same day were separately kept for incubating and first to fourth larvae were collected at the newly emerging stage (within 12 h).

### 2.3. RNA Extraction and Transcriptome Sequencing

According to the technical manual, each replicate had 30 mg of larval samples and the RNA of all insect samples (three biological replicates) was extracted using Invitro-gen™ TRIzol™ (Life Technologies, Carlsbad, CA, USA). Each replicate had 150 mg of leaf tissue samples and the RNA of all plant leaf samples was extracted using Eastep^®^ Super Total RNA Extraction Kit (Promega, Shanghai, China). The quality and quantity of RNA were assessed by measuring the absorbance using a UV spectrophotometer (GE Healthcare Bio-Science, Uppsala, Sweden) and the RNA integrity was further confirmed with 1% agarose gel electrophoresis. Transcriptome sequencing was conducted on an Illumina platform by Biomarker Technologies Company (Beijing, China) with high quality RNA samples to generate 150 bp paired-end reads. A total amount of 1 μg RNA per sample was used as the input material for each sample during the RNA preparation process. Sequencing libraries were constructed using the Hieff NGS Ultima Dual-mode mRNA Library Prep Kit for Illumina (Yeasen Bio-technology (Shanghai) Co., Ltd., Shanghai, China) following the manufacturer’s recommendations. The concentration of cDNA and the size of the inserts were assessed using Qubit 3.0 and Agilent 2100 to verify the library quality. The qualified libraries were sequenced on a high-throughput platform using the PE150 mode.

### 2.4. Transcriptome Data Analysis

Useless data were removed from the raw data before further analysis with inhouse perl scripts. Clean data were obtained by removing adapter-containing reads, Ploy-N-containing reads and low quality reads (reads with N ratio greater than 10%; reads with a quality value Q ≤ 10 accounting for more than 50% of the total readings).

HISAT2 (2.0.4) [17] was used to map the RNA-seq reads and StringTie (v2.2.1) [18] was applied to assemble the mapped reads. The clean reads were mapped to the genome of *S. lycopersicum* (*Solanum lycopersicum* ITAG2.4, https://phytozome-next.jgi.doe.gov/info/Slycopersicum_ITAG2_4) (accessed on 20 September 2024) [19] and the genome of *T. absoluta* (GCA_027580185.1_ASM2758018v1, https://www.ncbi.nlm.nih.gov/datasets/genome/GCA_027580185.1/) (accessed on 20 September 2024) [20]. Gene expression levels were estimated using FPKM values (fragments per kilobase of exon per million fragments mapped) by the StringTie (v2.2.1) [21]. The genes that were significantly differently expressed under different treatments or groups were defined as differentially expressed genes (DEGs). In this study, differential expression analysis was processed by DESeq2 (1.30.1) [22]. The criteria for differentially expressed genes was set as fold-change (FC) with an absolute value of ≥2 and FDR < 0.01. The DEGs were then subjected to functional enrichment analysis. The DEGs were identified in differential expression analysis and were annotated in COG, GO, KEGG, and KOG. The enriched GO terms and corresponding inclusion relationships are shown in the directed acyclic graph. The directed acyclic graphs of DEGs were generated by topGO [23,24].

### 2.5. Specific Gene Sequence Annotation

The sequences of the genes of interest were annotated using BLAST analysis in NCBI and the gene function was annotated based on the databases Nr, Pfam, KOG/COG, Swiss-Prot, KO, and GO. The corresponding phylogenetic trees were generated with relatives of arthropods using a neighbor-joining method with 1000 bootstrap replications implemented in MEGA 4.0 [25]. The conserved domains of the coding proteins were confirmed via CD-search in the NCBI database (https://www.ncbi.nlm.nih.gov/Structure/cdd/wrpsb.cgi) (accessed on 21 September 2024). Three-dimensional structures were generated using SWISS MODEL [26]. The interaction of proteins was predicted using the HDOCK SERVER [27] and visualized using Mol* software (https://www.rcsb.org/3d-view, accessed on 21 September 2024) [28]. All the software parameter settings are default.

## 3. Results

### 3.1. Sequencing Data Statistics

At least 5.95 Gb clean data were generated for each insect sample with a minimum 87.56% of clean data achieving a quality score of Q30 and the mapping ratio ranging from 73.96% to 80.98%. At least 6.04 Gb clean data were generated for each plant sample with a minimum 94.26% of clean data achieving a quality score of Q30 and the mapping ratio ranging from 72.20% to 97.38%. High quality data of the tested samples were generated via transcriptome analysis (Supplementary Table S1). In the response of *S. lycopersicum*, 1451 and 2971 DEGs were identified in the mechanical damage (M_vs_C) and feeding damage groups (F_vs_C) (Supplementary Figure S1). In M_vs_C, the expression levels of 905 genes were significantly up-regulated and 546 genes were down-regulated (Supplementary Table S2). For enrichment annotation, DEGs were classified in the COG, GO, KEGG, and KOG, and most genes (1168) were annotated in the GO database. In F_vs_C, the expression levels of 1823 genes were significantly up-regulated and 1148 genes were down-regulated (Supplementary Table S3). Most genes (2418) were annotated in the GO database as well. Details of the DEG data of the *S. lycopersicum* samples are available in Supplementary Table S4. The DEGs in the two comparison groups also showed specificity and overlap; there were 744 DEGs both identified in both groups (Supplementary Figure S2).

### 3.2. GO Enrichment of Response Genes to Mechanical and Feeding Damage

**Figure 1:**
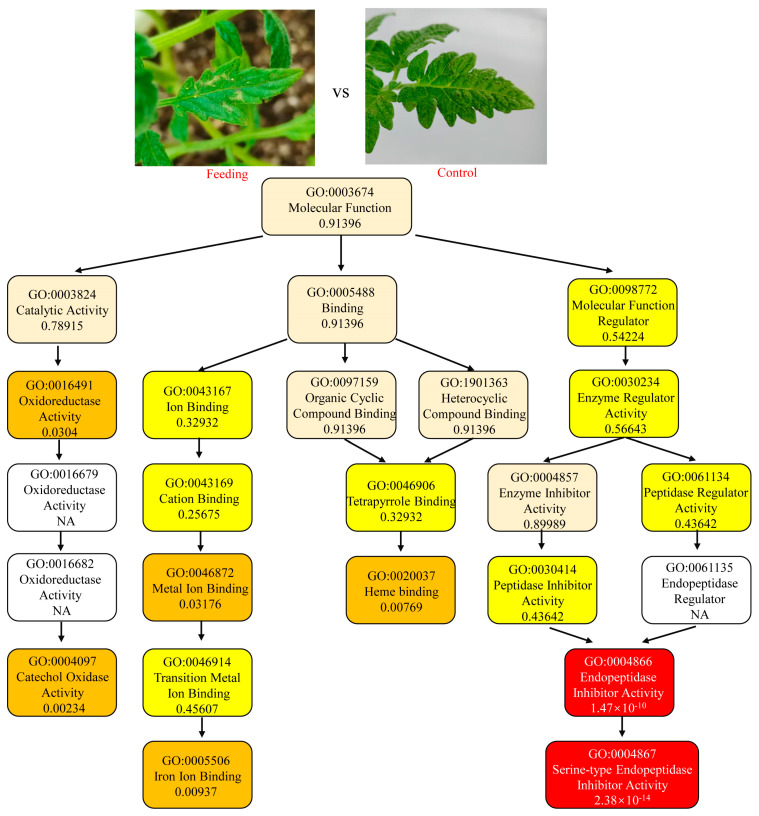
Top enrichment of DEGs in leaves of *Solanum lycopersicum* infested with *Tuta absoluta*. Enrichment was performed on each GO term under the molecular function, with the most significant nodes. Each box provides a description of the content and enrichment significance value of the GO term. Different colors represent different enrichment significance and the darker the color, the higher the significance.

**Figure 2:**
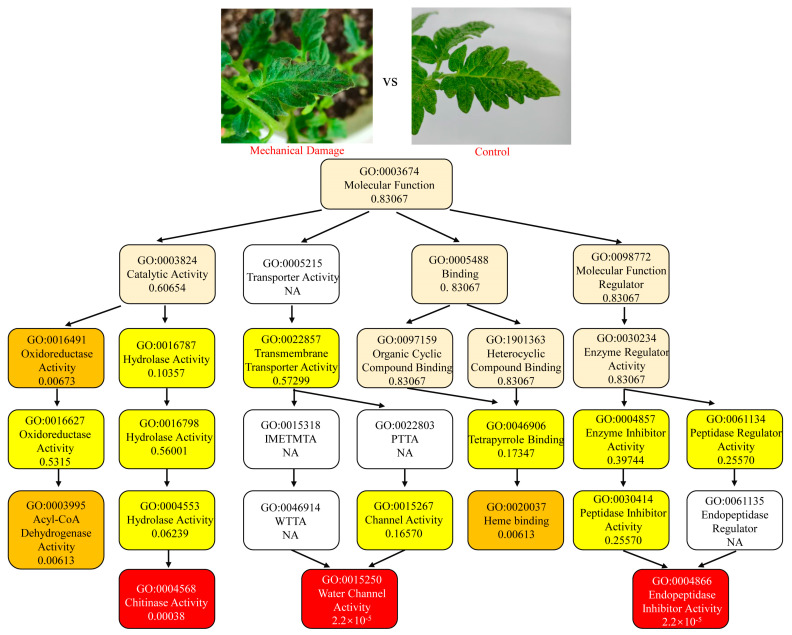
Top enrichment of DEGs in leaves of *Solanum lycopersicum* with mechanical damage. Enrichment was performed on each GO term under the molecular function, with the most significant nodes. Each box provides a description of the content and enrichment significance value of the GO term. Different colors represent different enrichment significance and the darker the color, the higher the significance.

In F_vs_C, Endopeptidase Inhibitor Activity (GO0004866) and Serine-type Endopeptidase Inhibitor Activity (GO0004867) were two most significantly enriched downstream GO terms, which indicated that the genes of *S. lycopersicum* with a function such as endopeptidase inhibitors worked in response to feeding by *T. absoluta* (Figure 1). In comparison, in M_vs_C, Chitinase Activity (GO0004568), Water Channel Activity (GO0015250), and Endopeptidase Inhibitor Activity (GO0004866) were the three most significantly enriched downstream GO terms. The differences showed that the responses of *S. lycopersicum* had specificity to insect feeding and mechanical damage (Figure 2). To provide a comprehensive view of the DEGs annotations, the enrichments of the top GO terms in Biological Process and Cellular Component are also provided in Supplementary Figures S3–S6.

### 3.3. Bioinformatics and Expression Analysis of the Endopeptidase Inhibitor Genes of S. lycopersicum

**Figure 3:**
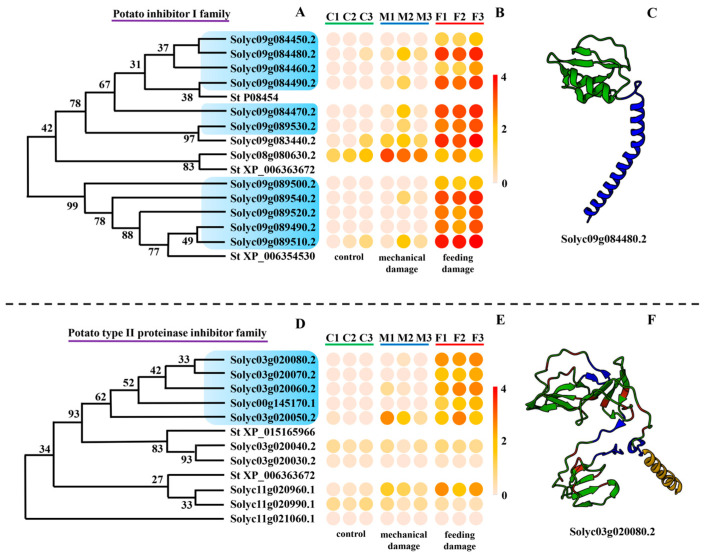
Molecular and expression information of potato inhibitor family genes. (**A**,**D**) Evolutionary tree of protein sequences of genes belonging to the potato inhibitor I (**A**) and potato type II proteinase inhibitor families (**D**). Genes specifically expressed in the feeding group are marked in blue. *St P08454*, *St XP_006363672*, *St XP_006354530*, *St XP_015165966*, and *St XP_006363672* are genes of *Solanum tuberosum*. (**B**,**E**) Expression response of the potato inhibitor I family (**B**) and potato type II proteinase inhibitor family gene (**E**) to mechanical damage and *Tuta absoluta* feeding. The expression data are based on Log10 (FPKM) of each treatment. (**C**,**F**) Protein structure of important response genes from the potato inhibitor I (**C**) and potato type II proteinase inhibitor families (**F**).

**Figure 4:**
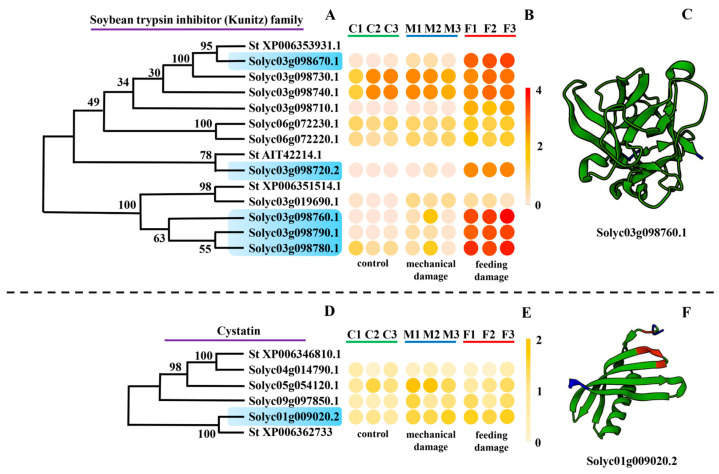
Molecular and expression information of the soybean trypsin inhibitor (Kunitz) family and aspartic acid proteinase inhibitor genes. (**A**,**D**) Evolutionary tree of protein sequences of genes belonging to the soybean trypsin inhibitor (Kunitz) (**A**) and cystatin families (**D**). Genes specifically expressed in the feeding group are marked in blue. *St XP006353931.1*, *St AIT42214.1*, *St XP006351514.1*, *St XP006346810.1*, and *St XP006362733* are genes of *Solanum tuberosum*. (**B**,**E**) Expression response of the soybean trypsin inhibitor (Kunitz) family (**B**) and cystatin family genes (**E**) to mechanical damage and *Tuta absoluta* feeding. The expression data are based on Log10 (FPKM) of each treatment. (**C**,**F**) Protein structure of important response genes from the soybean trypsin inhibitor (Kunitz) (**C**) and cystatin families (**F**).

Since the response of genes with endopeptidase inhibitor activity, especially serine-type endopeptidase inhibitor activity, to insect feeding was notable, all the expressed endopeptidase inhibitor genes were screened out from the transcriptome data of *S. lycopersicum*. In total 35 genes were annotated as serine-type endopeptidase inhibitors. These genes were divided into three families as the potato inhibitor I family (Figure 3A), potato type II proteinase inhibitor family (Figure 3D), and soybean trypsin inhibitor (Kunitz) family (Figure 4A).

#### 3.3.1. Potato Inhibitor I Family

There were 13 genes belonging to the potato inhibitor I family. The expression patterns of genes in this family were quite specific, most of them expressed only and highly in the feeding damage group (Figure 3B). Typical genes such as *Solyc09g084480.2* and *Solyc09g089540.2* almost did not express in the leaves of *S. lycopersicum* without any infection, showed a slight response to mechanical damage, and reacted intensely to feeding by *T. absoluta*. The proteins in this family had a conservative domain that was predicted, such as wound-induced proteinase inhibitor 1 interacting with both chymotrypsin and trypsin (Figure 3C).

#### 3.3.2. Potato Type II Proteinase Inhibitor Family

Nine genes were annotated as members of the potato type II proteinase inhibitor family (Figure 3D). Compared with genes from the potato inhibitor I family, the response of these genes to feeding by *T. absoluta* was not as strong, but some of them were also specifically expressed (Figure 3E). The expression patterns of *Solyc03g020080.2*, *Solyc03g020070.2*, *Solyc03g020060.2*, and *Solyc00g145170.1* only react to feeding by *T. absoluta*. The typical protein structure is shown in Figure 3F.

#### 3.3.3. Soybean Trypsin Inhibitor (Kunitz) Family

Kunitz-type protease inhibitors could protect the plant by inhibiting proteases of the invading organisms. Eleven genes belonging to the soybean trypsin inhibitor (Kunitz) family were identified (Figure 4A). Some of these genes maintained a high expression level in all samples. Meanwhile, there were still specific genes (*Solyc03g098670.1*, *Solyc03g098720.2*, *Solyc03g098760.1*, *Solyc03g098780.1*, *Solyc03g098790.1*) with a strong reaction to feeding (Figure 4B). The proteins of this family had a conservative domain and the typical protein structure is shown in Figure 4C.

#### 3.3.4. Cystatin Family

Genes belonging to the cystatin family function as inhibitors of cysteine proteases. Four genes were classified in this family (Figure 4D). Only *Solyc01g009020.1* showed an apparent reaction to both feeding damage and mechanical damage (Figure 4E). The typical protein structure is shown in Figure 4F.

### 3.4. Bioinformatics and Expression Analysis of the Endopeptidase Genes of T. absoluta

The transcriptome data of different development stages of *T. absoluta* larvae were also sequenced to identify highly expressed endopeptidase genes, which might be the potential interacting targets of these inhibitors. In total, five genes were annotated as cysteine protease, and thirty-one genes were annotated as serine protease.

#### 3.4.1. Expression of Genes Encoding Cysteine Proteases in the Developmental Stages of *T. absoluta* Larvae

**Figure 5:**
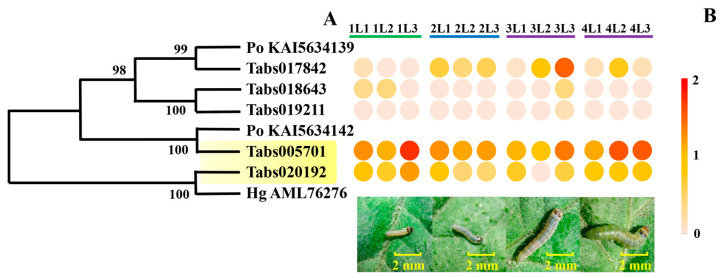
Molecular and expression information of cysteine peptidase genes during larvae development of *Tuta absoluta*. (**A**) Evolutionary tree of protein sequences of the cysteine peptidase genes. (**B**) Expression response of the cysteine peptidase genes during larvae development of *Tuta absoluta*. The expression data are based on Log10 (FPKM) of each treatment. Genes with high expression levels during all the developmental stages are marked in yellow. *Po KAI5634139* and *Po KAI5634142* are cysteine peptidase genes of *Phthorimaea operculella*. *Hg AML76276* is a gene of *Helicoverpa armigera*.

The expression of five genes encoding cysteine proteases was detected in the transcriptome data of *T. absoluta* larvae (Figure 5A). Four of them (*Tabs017842*, *Tabs018643*, *Tabs019211*, *Tabs005701*) encode proteins belonging to the C1 family peptidase and *Tabs0020192* encodes protein belonging to another family called the peptidase family C54. *Tabs005701* and *Tabs0020192* had high expression levels during the developmental stages of *T. absoluta* larvae (Figure 5B).

#### 3.4.2. Expression of Genes Encoding Serine Proteases in the Developmental Stages of *T. absoluta* Larvae

**Figure 6:**
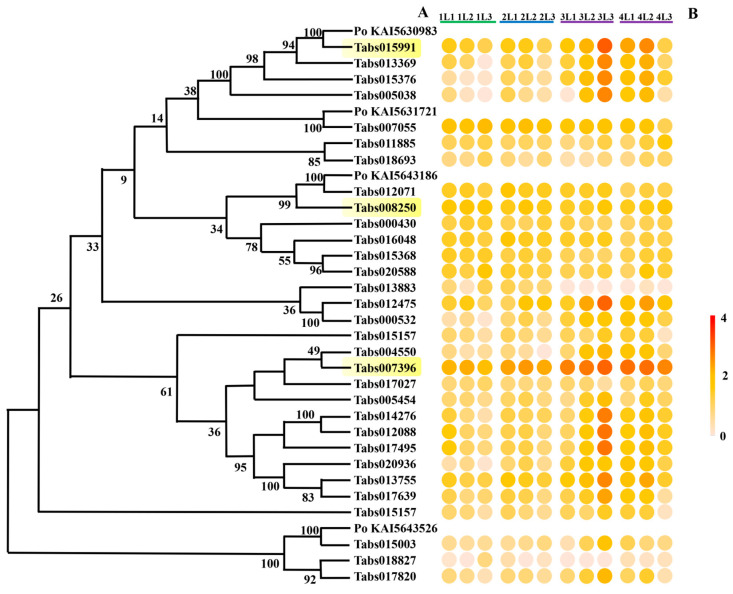
Molecular and expression information of serine protease genes during the larvae development of *Tuta absoluta*. (**A**) Evolutionary tree of protein sequences of serine protease genes. *Po KAI5630983*, *Po KAI5631721*, *KAI5643186*, and *KAI5643526* are serine peptidase genes of *Phthorimaea operculella*. Genes with high expression levels during all the developmental stages are marked in yellow. (**B**) Expression response of serine protease genes during larvae development of *Tuta absoluta*. The expression data are based on Log10 (FPKM) of each treatment.

The expression of 31 genes encoding serine proteases was detected in the transcriptome data of *T. absoluta* larvae (Figure 6A). Three of them (*Tabs015003*, *Tabs018827*, *Tabs017820*) belong to the alpha/beta hydrolase family, which may catalyze the hydrolysis of substrates with different chemical compositions or physicochemical properties. The others belong to the Tryp_SPc superfamily with a trypsin-like domain. Most of these genes had a constantly high level of expression during the development of larvae (Figure 6B). Among these serine proteases, Tabs008250 and Tabs007396 were selected as representatives to predict their interactions with protease inhibitors of *S. lycopersicum*.

### 3.5. Prediction of Interactions Between Protease Inhibitors of S. lycopersicum and Proteases of T. absoluta

The typical protein structures of protease inhibitors and proteases were constructed using SWISS-MODEL and their binding mode was predicted using HDOCK SERVER. Using proteases as the receptor and protease inhibitors as the ligand, binding sites between specifically responding serine protease inhibitors of *S. lycopersicum* and important serine protease in *T. absoluta* larvae were predicted.

#### 3.5.1. Interactions Between Serine Inhibitors of *S. lycopersicum* and Serine Protease

Tabs008250 is a trypsin-like serine protease with a regulatory CLIP domain (Cys22-Cys74) and a secreted trypsin-like serine protease domain (Ala116-Ser378).

As a member of Potato type II protease inhibitors, Solyc03g020080.2 has two Prot_inhib_II domains (Lys32-Glu81, Arg89-141Gly). Five binding sites between Solyc03g020080.2 and Tabs008250 were identified (Supplementary Figure S7A).

Solyc09g084480.2 is a serine protease inhibitor with a typical domain (Lys48-Val110) belonging to the potato inhibitor I family. Four binding sites between Solyc09g084480.2 and Tabs008250 were identified (Supplementary Figure S7B).

Solyc03g098760.1 is a Kunitz-type protease inhibitor with a typical domain (Pro39-Lys200) belonging to the soybean trypsin inhibitor family. Three binding sites between Solyc03g098760.1 and Tabs008250 were identified (Supplementary Figure S7C).

These interacting sites were all within typical domains on protease or protease inhibitors.

#### 3.5.2. Interactions Between Serine Inhibitors of *S. lycopersicum* and Serine Protease Tabs007396 of *T. absoluta*

Tabs007396 represents another type of trypsin-like serine protease with a single domain of secreted trypsin-like serine protease (Leu8-Ala259).

Four binding sites between Solyc09g084480.2 and Tabs007396 were identified (Supplementary Figure S8A). The interaction sites on Solyc09g084480.2 were Ser83 (bound to Asp186 on Tabs007396), Val85 (bound to Gly235), Ala87 (bound to Val211, Ser213&233), and Phe89 (bound to Ser52).

Three binding sites between Solyc03g020080.2 and Tabs007396 were identified (Supplementary Figure S8B). The interaction sites on Solyc03g020080.2 were Ala48 (bound to Phe74 on Tabs007396), Lys110 (bound to Asp101), and Pro146 (bound to Ser109 and Ala148).

Two binding sites between Solyc03g098760.1 and Tabs007396 were identified (Supplementary Figure S8C). The interaction sites on Solyc03g098760.1 were Tyr72, (bound to Tyr62&75, and His102 on Tabs007396), and Ser122 (bound to Asp255).

#### 3.5.3. Interactions Between Cysteine Inhibitors of *S. lycopersicum* and Cysteine Protease Tabs005701 of *T. absoluta*

Tabs005701 is a cysteine proteinase belonging to the C1 family peptidase with a cysteine protease domain (Glu246-Thr579).

Solyc01g009020.2 is an aspartic acid proteinase inhibitor belonging to a family of cysteine protease inhibitors with a cystatin-like domain (Gly20-Lys105). Three binding sites between Solyc01g009020.2 and Tabs005701 were identified (Supplementary Figure S8D).

## 4. Discussion

Plants are often attacked by insect pests in nature. In response, plants have developed defense systems against herbivores. Insect behaviors including feeding, laying eggs, and defecating can trigger the defense response of host plants [29,30]. Attacks by *T. absoluta* activate various defense responses. The activity of polyphenol oxidase (PPO) in the damaged leaves is enhanced [31] and the important genes of cell wall metabolism are up-regulated to reshape cells and strengthen the external barrier [32]. Phenylpropanoid and polyamine precursor pathways are activated, accumulating a large amount of phenolamides in the affected leaves [33]. Protease inhibitors (PIs) are considered important factors in the plant defense system. Transcriptome data showed that 17 PI genes of a tolerant genotype tomato were specifically expressed after an attack by *T. absoluta* [32]. However, our study found that there are 35 genes encoding serine endopeptidase inhibitors with overexpression in the feeding group. These genes belong to three families, the potato inhibitor I, potato protease inhibitor II, and soybean trypsin inhibitor (Kunitz) families.

Potato inhibitor I (Pin I) was the first identified potato inhibitor family [34] and homologous sequences were later found in other plants, such as tomato and sweet potato [35,36]. Different from other inhibitors, proteins in Pin I only have one disulfide bond or do not have disulfide bonds, but rely on a large number of hydrogen bonds to stabilize the extended conformation of the reaction site loop [37,38]. Pin I PIs have been proven to be related to plant defense. After aphids or cabbage looper fed on potato plants, the potato type I protease inhibitor was up-regulated in the local leaves [39,40].

The protease inhibitors of potato type II (Pin II PIs) are the most widely characterized plant PI and the interaction between Pin II and protease is determined by the reaction center loop (RCL), which is a tripeptide region with two highly conserved cysteine residues on both sides. The RCL tripeptide is very important for the specificity and efficiency of inhibitors [41]. This structure causes Pin II PIs to have a good inhibitory activity on serine protease-like enzymes in the intestine of *Helicoverpa armigera* larvae [42].

Kunitz-type protease inhibitors are abundant in leguminous plants and are considered to play a role in storing nutrients and defending against insect predation [43]. A protease inhibitor homologous to soybean Kunitz inhibitor in *Cicer arietinum* can affect the growth and development of cotton bollworm [44]. In the soybean trypsin inhibitor (Kunitz) family, the number of disulfide bonds is not fixed and many members have disulfide bonds between Cys39 and Cys86, which can stabilize the reaction center site [45,46]. In addition, the reduction degree of disulfide bonds is also related to the inhibitory activity of the inhibitor [47].

Proteolytic enzymes of insects can decompose proteins in food and these enzymes are mainly synthesized in the midgut. The composition of protease in the insect intestinal tract changes at different development stages according to the differences in food intake and digestion. Cysteine proteases were the main proteolytic enzyme expressed in the first larvae of *Anticarsia gemmatalis*, while in the fifth larvae, the main proteolytic enzyme changed to serine proteases [48]. High expressions of serine proteases were also detected in the third to fifth larvae of *H. armigera* [49]. Trypsin exists widely in all kinds of insects and the gene functions of this family are diverse, mainly related to food digestion, immune defense response, nervous system, and insecticide resistance [50,51,52]. Trypsin is an endoprotease that mainly cuts the peptide chain on the carboxyl side of the amino acid lysine or arginine and 97% of the bonds formed between the peptide and trypsin are hydrogen bonds [53].

PIs are very important in the plant defense system and the insect control based on PIs depends on the expression of PIs. The Kunitz-type genes and Pin I genes from potato were expressed in *Nicotiana benthamiana* plants and showed resistance to fungi [54]. A Pin II gene in pepper was introduced into tomato plants and overexpressed, making the transgenic tomatoes resistant to *H. armigera* [55]. PIs also worked through feeding to insects. A constitutive serine protease inhibitor can inactivate the digestive proteases of *Ectropis obliqua* and *Spodoptera frugiperda* by feeding [56]. The mixed use of different types of inhibitors may have a better insecticidal effect [51]. A potato type I protease inhibitor and a type II inhibitor were co-expressed in transgenic cotton and the cotton showed high resistance to *Helicoverpa punctigera* [57]. Heterologous expression of PI genes in non-preferred host plants is a hot research topic at present and may be the main application route of serine protease inhibitors in the future.

In our study, the changes in enzymes caused by insect feeding and mechanical damage showed both specificity and overlap, which means there is both chemical stimulation and mechanical damage in the process of insect feeding on plants. Studies have shown that, compared with mechanical damage, *Phthorimaea operculella* feeding has a greater impact on the biosynthesis of potato serine protease inhibitors [58]. Salivary compounds of insects are important factors that can induce specific defense responses in different host plants other than mechanical damage.

## 5. Conclusions

In this study, serine-type endopeptidase inhibitors were characterized as important response factors in *S. lycopersicum* to resist feeding by *T. absoluta*, specific serine-type endopeptidase inhibitor genes were screened out and their potential interacting targets in *T. absoluta* were predicted. These data are helpful to develop new pest control compounds based on the interaction between host and pest.

## Data Availability

The datasets generated and analyzed during the current study are included in this article. The sequencing data that support the findings of this study are openly available in the Phytozome database (https://phytozome-next.jgi.doe.gov/info/Slycopersicum_ITAG2_4) (accessed on 20 September 2024). Raw reads for RNA-Seq are available from the CNCB database (https://ngdc.cncb.ac.cn/gsa/) (accessed on 20 September 2024) with accession number CRA014589 and CRA014590.

## Supplementary Materials

The following supporting information can be downloaded at: https://www.mdpi.com/article/10.3390/insects16020166/s1, Figure S1: Venn diagram of DEGs between two comparison groups; Figure S2: Up-regulated DEGs enrichment in GO; Figure S3: Up-regulated DEGs of M_vs_C enrichment in Biological Process; Figure S4: Up-regulated DEGs of M_vs_C enrichment in Cellular Component; Figure S5: Up-regulated DEGs of F_vs_C enrichment in Biological Process; Figure S6: Up-regulated DEGs of F_vs_C enrichment in Cellular Component; Figure S7: Interaction mode between proteinase inhibitors from *Solanum lycopersicum* and pro-teinase from *Tuta absoluta* (Solyc03g020080.2, Solyc09g084480.2, Solyc03g098760.1) and pro-teinase from *Tuta absoluta* (Tabs008250); Figure S8: Interaction mode between proteinase inhibitors from *Solanum lycopersicum* (Solyc09g084480.2, Solyc03g020080.2, Solyc03g098760.1, Solyc01g009020.2) and proteinases from *Tuta absoluta* (Tabs007396, Tabs005701); Table S1: Summary statistics of samples; Table S2: DEGs in M_vs_C with details of expression and annotation; Table S3: DEGs in F_vs_C with details of expression and annotation; Table S4: Statistics of DEGs of *Solanum lycopersicum* samples.
