# Supplementary material for: Comparative Transcriptome Analysis Reveals Expression of Defense Pathways and Specific Protease Inhibitor Genes in Solanum lycopersicum in Response to Feeding by Tuta absoluta
Source: Insects. 2025 Feb 5;16(2):166. doi: 10.3390/insects16020166 (PMC11855745; doi:10.3390/insects16020166)
Supplement: Supplementary file 1 [file insects-16-00166-s001.zip › Supplementary Figure captions.pdf]

Supplementary Figure S1 Venn diagram of DEGs between two comparison groups

Green: DEGs in Feeding damage group_vs_Control group

Blue: DEGs in Mechanical damage group_vs_Control group

Supplementary Figure S2 Up-regulated DEGs enrichment in GO

F vs C: Feeding damage group_vs_Control group

M vs C: Mechanical damage group_vs_Control group

Supplementary Figure S3 Up-regulated DEGs of M_vs_C enrichment in Biological Process

Supplementary Figure S4 Up-regulated DEGs of M_vs_C enrichment in Cellular Component

Supplementary Figure S5 Up-regulated DEGs of F_vs_C enrichment in Biological Process

Supplementary Figure S6 Up-regulated DEGs of F_vs_C enrichment in Cellular Component

Supplementary Figure S7 Interaction mode between proteinase inhibitors from *Solanum lycopersicum* and pro-teinase from *Tuta absoluta* (Solyc03g020080.2, Solyc09g084480.2, Solyc03g098760.1) and pro-teinase from Tuta absoluta (Tabs008250).

For interaction prediction, proteinases from *Tuta absoluta* are set as Receptors and labeled in orange, proteinase inhibitors from *Solanum lycopersicum* are set as ligands and labeled in green. (A) Interaction mode between Solyc03g020080.2 and Tabs008250. (B) Interaction mode between Solyc09g084480.2 and Tabs008250. (C) Interaction mode between Solyc03g098760.1 and Tabs008250.

Supplementary Figure S8 Interaction mode between proteinase inhibitors from *Solanum lycopersicum* (Solyc09g084480.2, Solyc03g020080.2, Solyc03g098760.1, Solyc01g009020.2) and proteinases from *Tuta absoluta* (Tabs007396, Tabs005701).

For interaction prediction, proteinases from *Tuta absoluta* are set as Receptors and labeled in orange, proteinase inhibitors from *Solanum lycopersicum* are set as ligands and labeled in green. (A) Interaction mode between Solyc03g020080.2 and Tabs008250. (B) Interaction mode between Solyc09g084480.2 and Tabs008250. (C) Interaction mode between Solyc03g098760.1 and Tabs008250.
