# Supplementary material for: Comparative Transcriptome Analysis Reveals Expression of Defense Pathways and Specific Protease Inhibitor Genes in Solanum lycopersicum in Response to Feeding by Tuta absoluta
Source: Insects. 2025 Feb 5;16(2):166. doi: 10.3390/insects16020166 (PMC11855745; doi:10.3390/insects16020166)
Supplement: Supplementary file 1 [file insects-16-00166-s001.zip › Supplementary table S1.pdf]

Supplementary Table S1 Summary statistics of samples

Samples	Clean reads (Pair ends)	%≥Q30	Mapped Reads (Single end)	Mapping Ratio
C1	20,236,541	95.27%	39,383,291	97.31%
C2	20,166,043	94.72%	39,275,631	97.38%
C3	21,411,452	95.02%	41,501,864	96.92%
M1	20,686,051	95.12%	40,193,925	97.15%
M2	20,255,249	95.08%	37,598,559	92.81%
M3	21,091,304	94.26%	40,918,376	97.00%
F1	20,291,693	95.01%	30,682,466	75.60%
F2	20,397,798	95.19%	29,455,745	72.20%
F3	20,807,035	95.85%	34,166,241	82.10%
L1-1	20,384,134	88.69%	31,879,759	78.20%
L1-2	20,129,825	88.76%	32,011,995	79.51%
L1-3	19,865,754	87.56%	29,956,680	75.40%
L2-1	20,370,761	89.38%	31,886,689	78.27%
L2-2	20,181,908	88.98%	31,587,846	78.26%
L2-3	20,331,521	88.80%	32,150,585	79.07%
L3-1	20,528,415	88.93%	30,365,144	73.96%
L3-2	20,052,806	88.50%	32,475,993	80.98%
L3-3	21,483,112	88.48%	33,074,536	76.98%
L4-1	20,424,887	87.72%	32,534,291	79.64%
L4-2	20,449,657	88.67%	31,642,659	77.37%
L4-3	20,348,104	88.78%	32,373,455	79.55%

Note: C1-3, three replicates of control of *S. lycopersicum* leaves

M1-3, three replicates of mechanical damage of *S. lycopersicum* leaves

F1-3, three replicates of feeding damage of *S. lycopersicum* leaves

L1-1, 2, 3, three replicates of first larvae of *T. absoluta*

L2-1, 2, 3, three replicates of second larvae of *T. absoluta*

L3-1, 2, 3, three replicates of third larvae of *T. absoluta*

L4-1, 2, 3, three replicates of fourth larvae of *T. absoluta*
