# Supplementary material for: Comparative Transcriptome Analysis Reveals Expression of Defense Pathways and Specific Protease Inhibitor Genes in Solanum lycopersicum in Response to Feeding by Tuta absoluta
Source: Insects. 2025 Feb 5;16(2):166. doi: 10.3390/insects16020166 (PMC11855745; doi:10.3390/insects16020166)
Supplement: Supplementary file 1 [file insects-16-00166-s001.zip › Supplementary table S4.pdf]

Supplementary table S2 Statistics of DEGs of *Solanum lycopersicum* samples

DEG Set	DEG Number	up-regulated	down-regulated	COG	GO	KEGG	KOG
C vs M	1,451	905	546	568	1168	995	742
C vs F	2,971	1,823	1,148	1113	2418	2050	1620

Note: C, Control group

M, Mechanical damage group

F, Feeding damage group
