# Supplementary figures and images for: Comparative Transcriptome Analysis Reveals Expression of Defense Pathways and Specific Protease Inhibitor Genes in Solanum lycopersicum in Response to Feeding by Tuta absoluta
Source: Insects. 2025 Feb 5;16(2):166. doi: 10.3390/insects16020166 (PMC11855745; doi:10.3390/insects16020166)

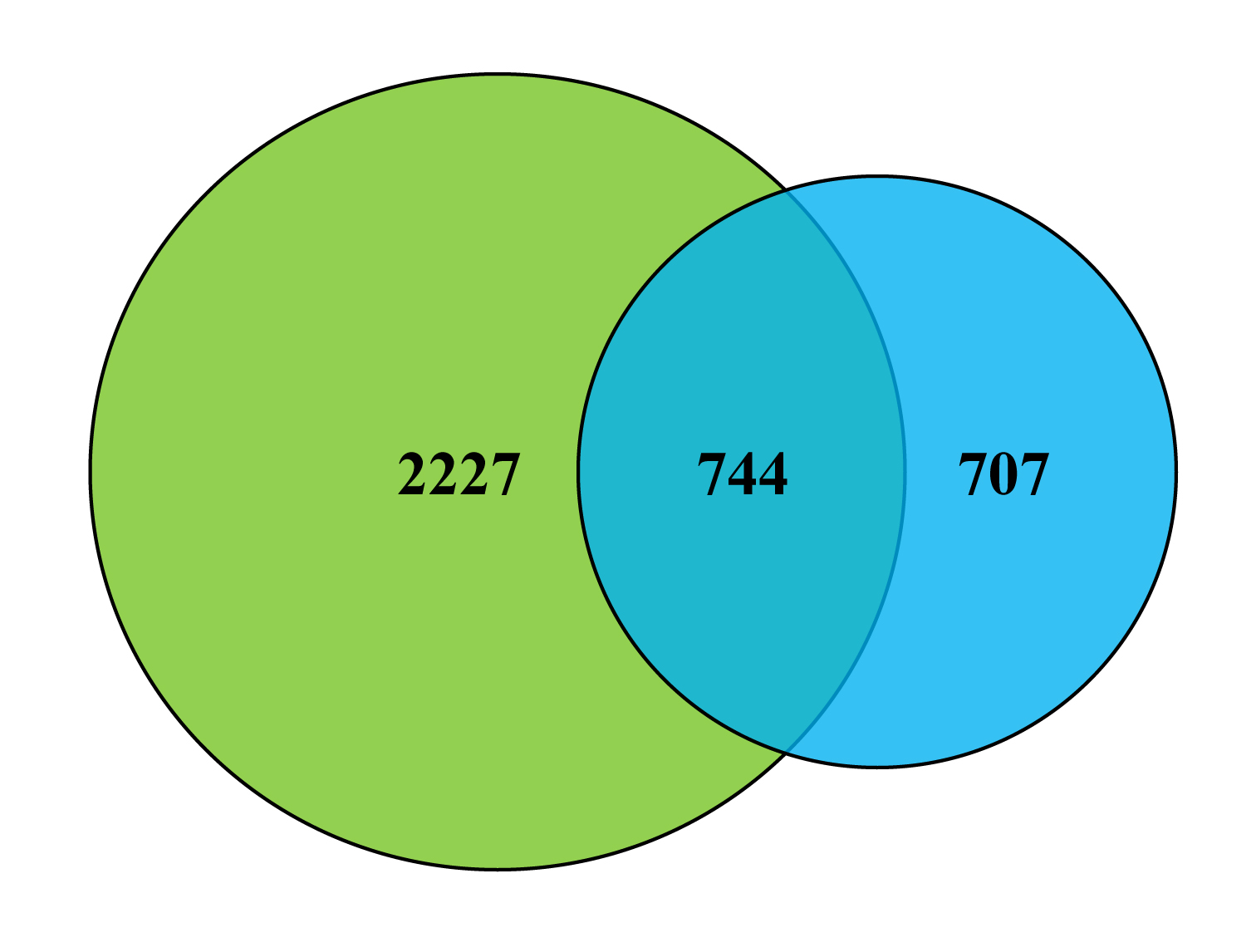

Supplement: Supplementary file 1 [file insects-16-00166-s001.zip › Supplementary Figure S1.jpg]

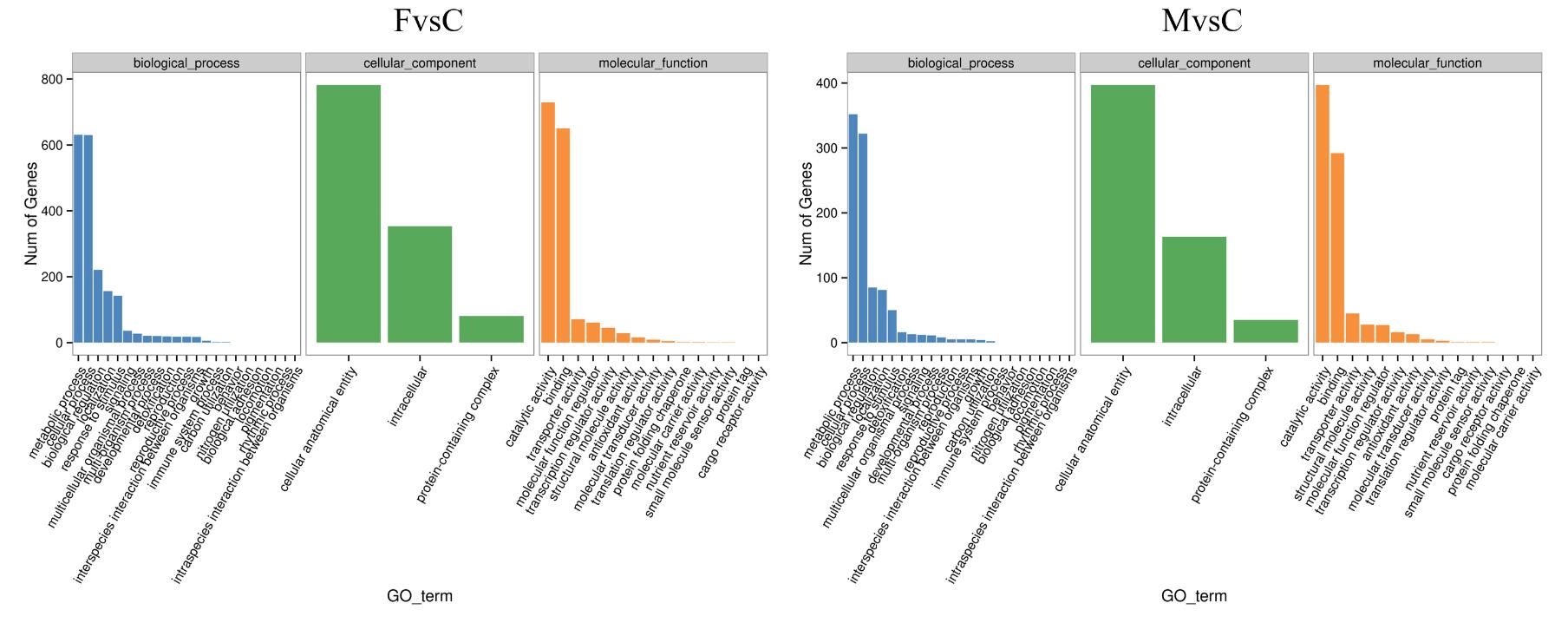

Supplement: Supplementary file 1 [file insects-16-00166-s001.zip › Supplementary Figure S2.jpg]

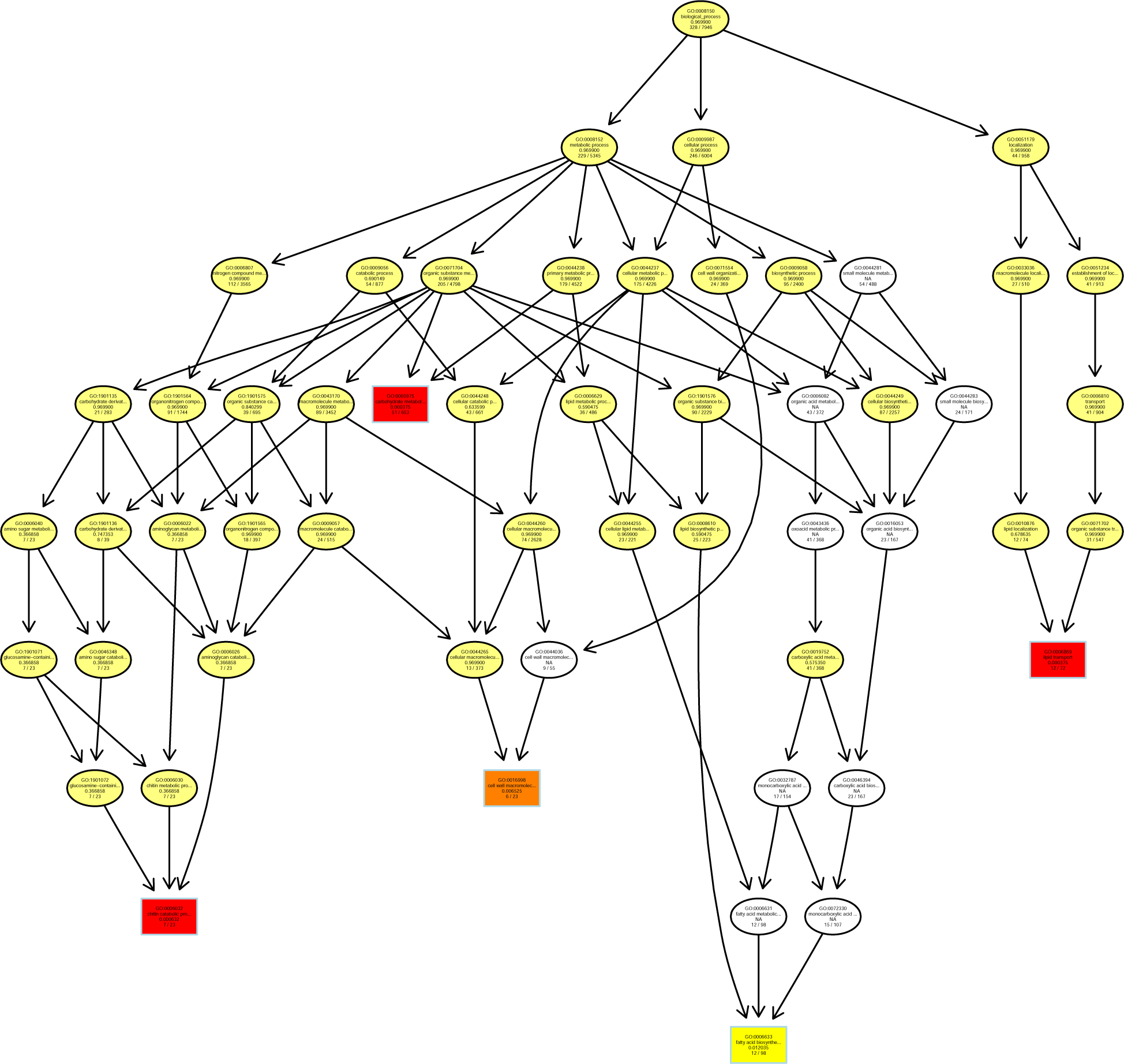

Supplement: Supplementary file 1 [file insects-16-00166-s001.zip › Supplementary Figure S3.jpg]

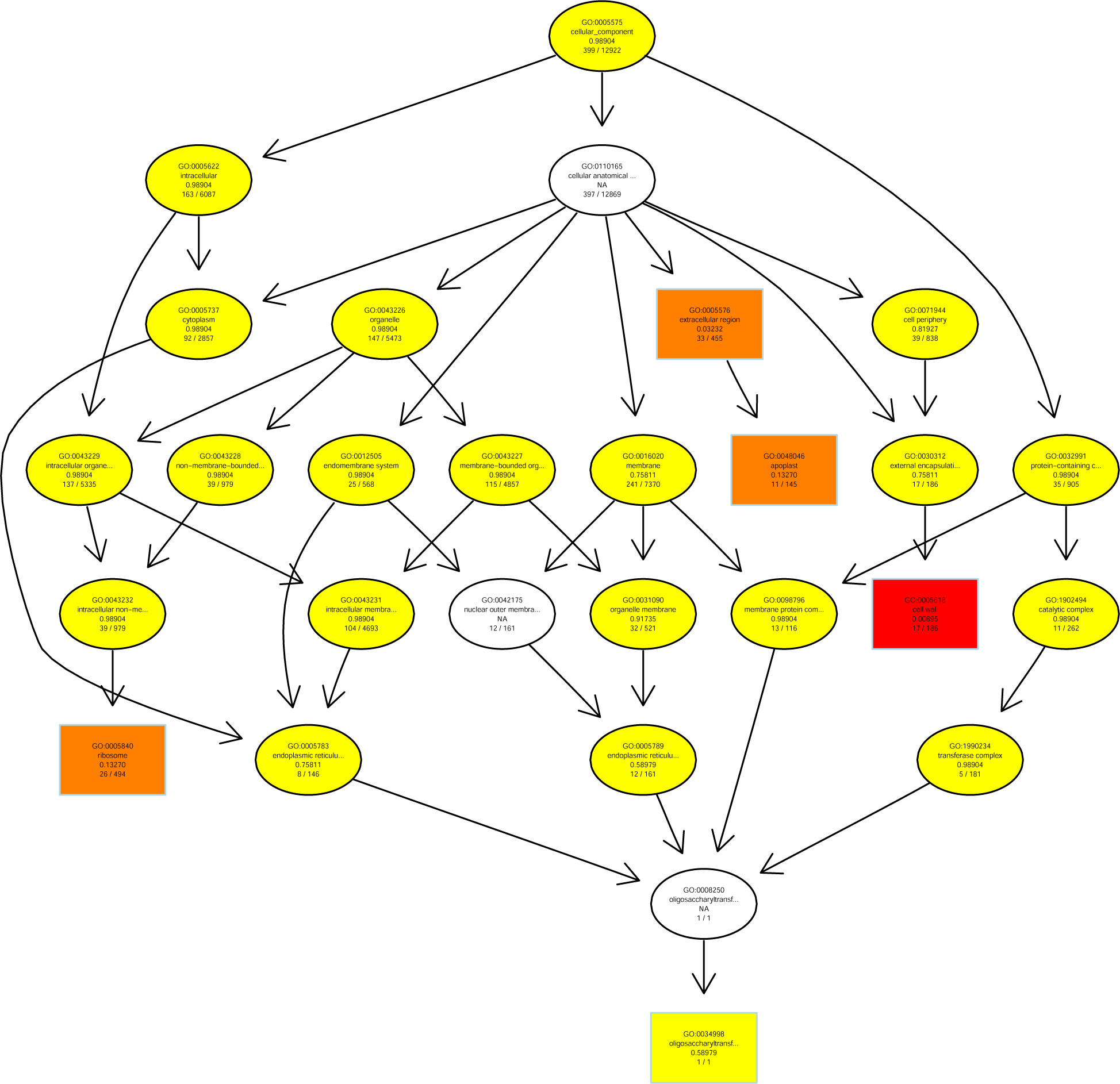

Supplement: Supplementary file 1 [file insects-16-00166-s001.zip › Supplementary Figure S4.jpg]

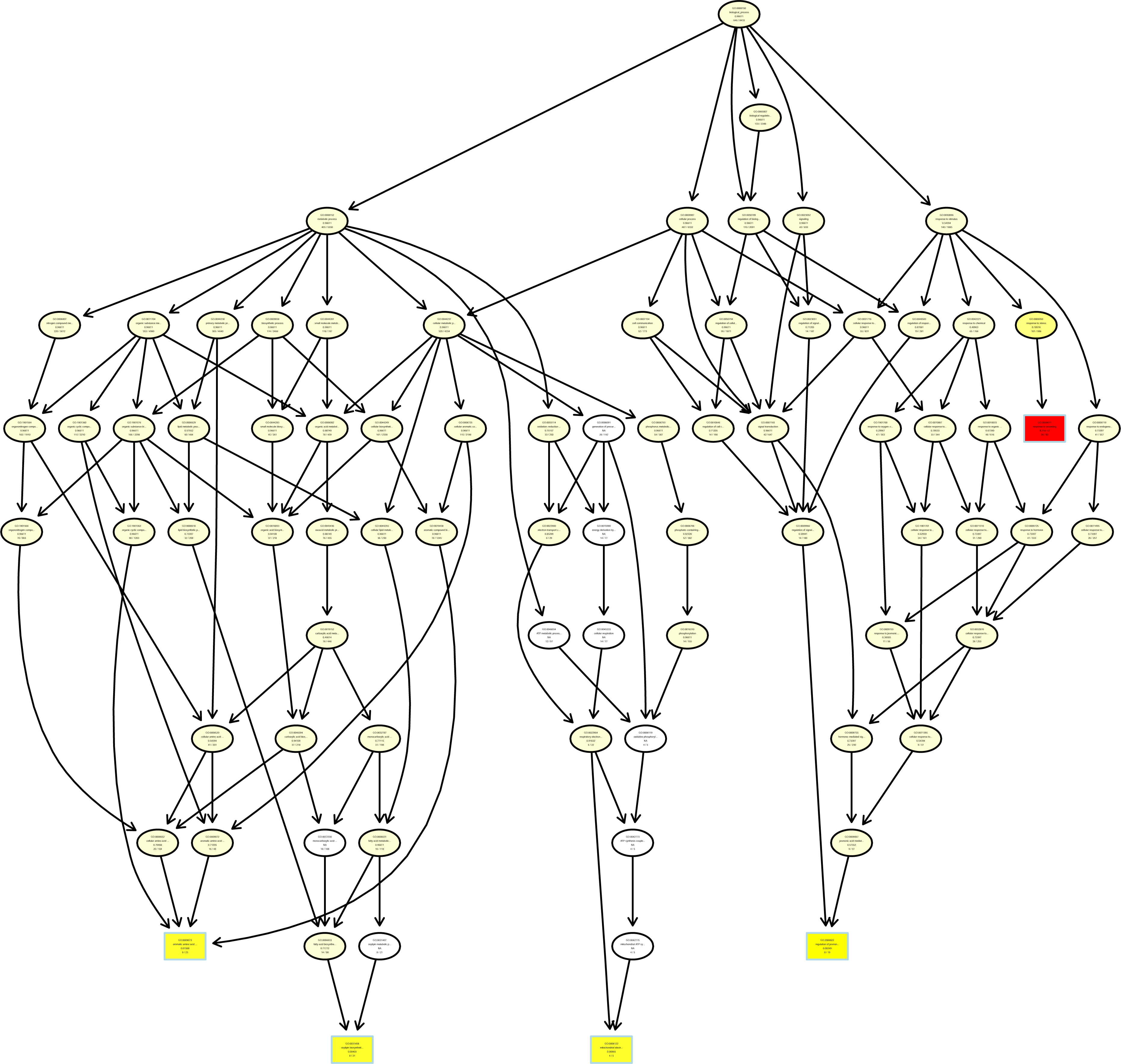

Supplement: Supplementary file 1 [file insects-16-00166-s001.zip › Supplementary Figure S5.jpg]

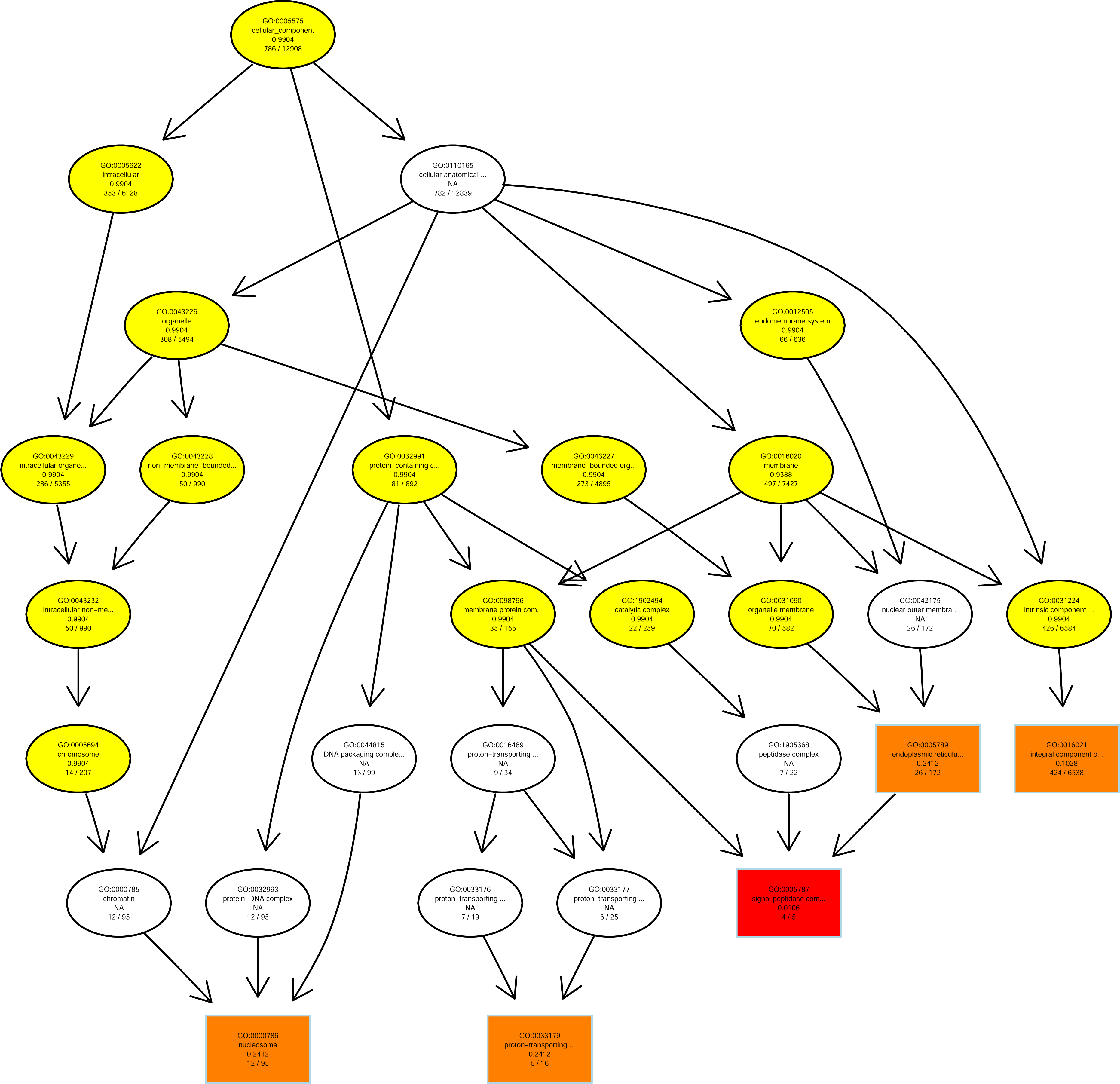

Supplement: Supplementary file 1 [file insects-16-00166-s001.zip › Supplementary Figure S6.jpg]

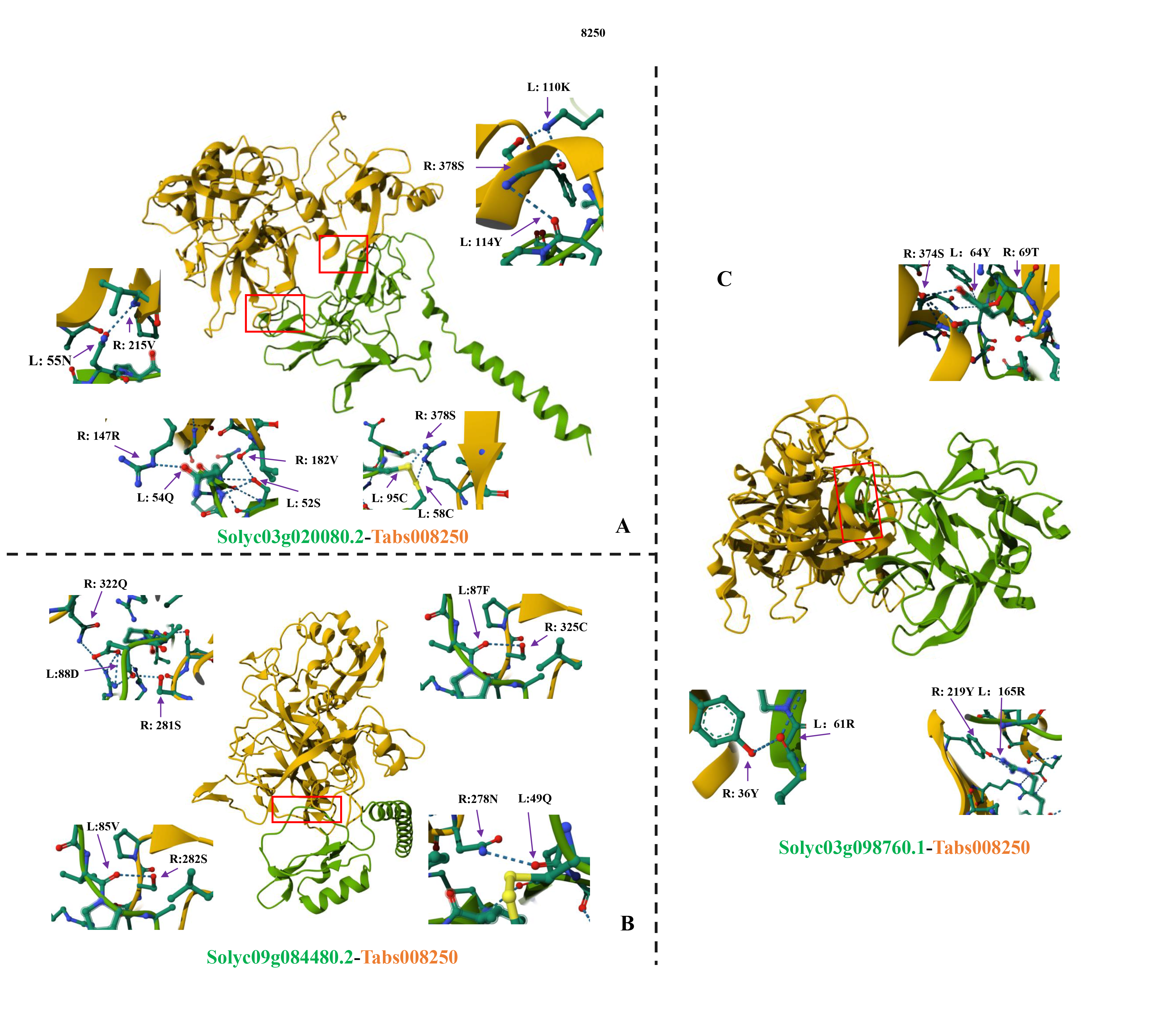

Supplement: Supplementary file 1 [file insects-16-00166-s001.zip › Supplementary Figure S7.png]

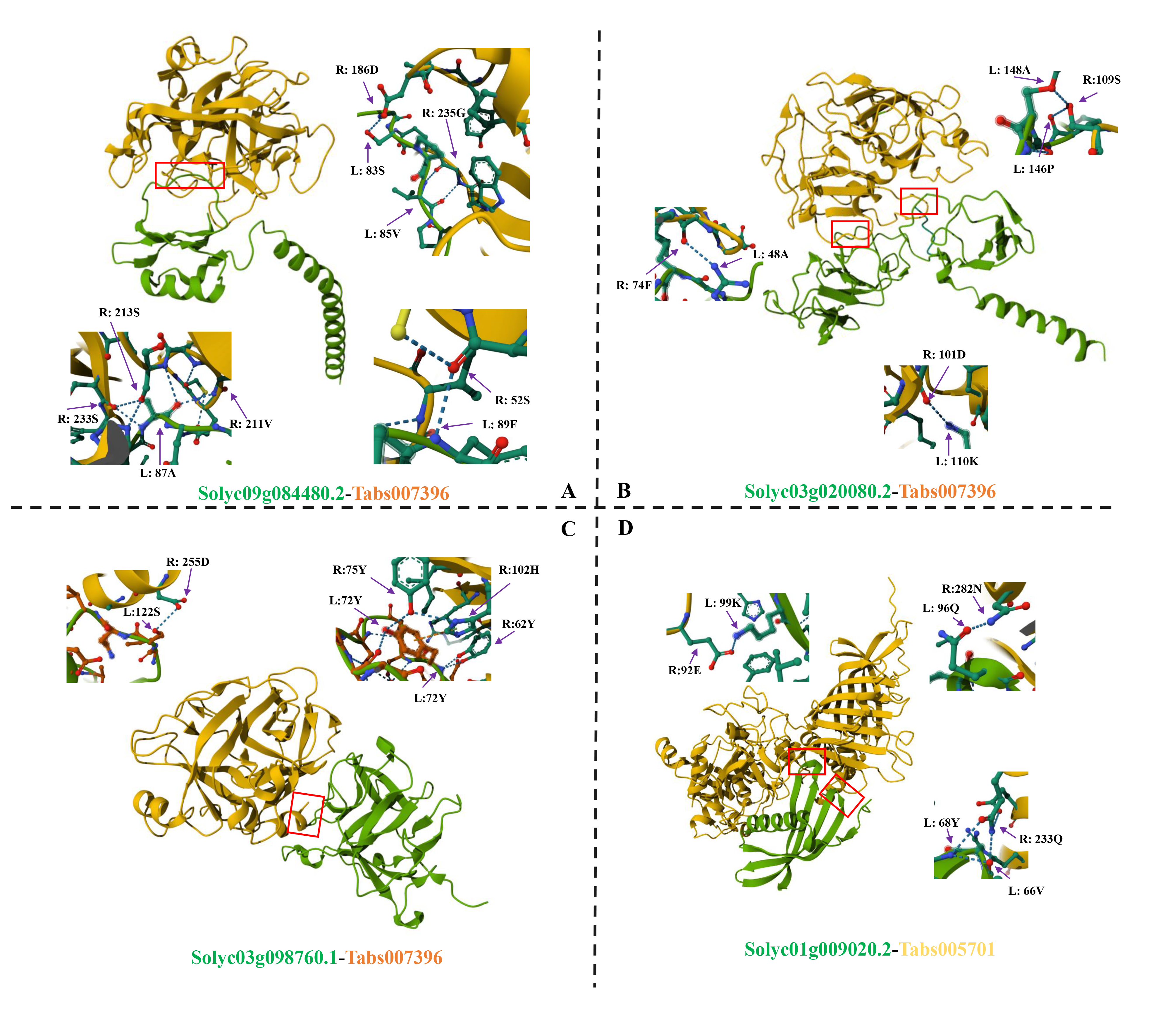

Supplement: Supplementary file 1 [file insects-16-00166-s001.zip › Supplementary Figure S8.png]
